# Human gut bacteria as potent class I histone deacetylase inhibitors *in vitro* through production of butyric acid and valeric acid

**DOI:** 10.1371/journal.pone.0201073

**Published:** 2018-07-27

**Authors:** Samantha Yuille, Nicole Reichardt, Suchita Panda, Hayley Dunbar, Imke E. Mulder

**Affiliations:** 4DPharma Research Ltd., Aberdeen, United Kingdom; Southern Illinois University School of Medicine, UNITED STATES

## Abstract

Overexpression of histone deacetylase (HDAC) isoforms has been implicated in a variety of disease pathologies, from cancer and colitis to cardiovascular disease and neurodegeneration, thus HDAC inhibitors have a long history as therapeutic targets. The gut microbiota can influence HDAC activity via microbial-derived metabolites. While HDAC inhibition (HDI) by gut commensals has long been attributed to the short chain fatty acid (SCFA) butyrate, the potent metabolic reservoir provided by the gut microbiota and its role in host physiology warrants further investigation in a variety of diseases. Cell-free supernatants (CFS) of 79 phylogenetically diverse gut commensals isolated from healthy human donors were screened for their SCFA profile and their total HDAC inhibitory properties. The three most potent HDAC inhibiting strains were further evaluated and subjected to additional analysis of specific class I and class II HDAC inhibition. All three HDAC inhibitors are butyrate producing strains, and one of these also produced substantial levels of valeric acid and hexanoic acid. Valeric acid was identified as a potential contributor to the HDAC inhibitory effects. This bacterial strain, *Megasphaera massiliensis* MRx0029, was added to a model microbial consortium to assess its metabolic activity in interaction with a complex community. *M*. *massiliensis* MRx0029 successfully established in the consortium and enhanced the total and specific HDAC inhibitory function by increasing the capacity of the community to produce butyrate and valeric acid. We here show that single bacterial strains from the human gut microbiota have potential as novel HDI therapeutics for disease areas involving host epigenetic aberrations.

## Introduction

The gut microbiota has been shown to play a prominent role in health and disease as an increasing number of diseases are linked to functional changes associated with an altered gut microbiota [1]. Apart from gastrointestinal diseases, such as IBS, IBD and colon cancer [2–5], recent studies have implicated gut bacteria in mucosal and systemic immune function, nutrition and obesity, cardiovascular diseases, liver function (gut-liver axis), diabetes (type 1 and type 2) (gut-pancreas axis), and brain function (gut-brain axis) [6–12].

Gut commensal communities and their hosts share a symbiotic relationship in which complex microbe-host and microbe-microbe communication is transmitted through a large variety of chemical signals, such as metabolites, small molecules, peptides, secreted and surface-associated proteins [1, 13–16].

One mechanism by which gut microbes are thought to initiate beneficial effects in the host is via their principal fermentation products, the short-chain fatty acids (SCFAs) acetate, propionate and butyrate. In the human gut, SCFAs reach total luminal concentrations of 50–200 mM, where primarily butyrate serves as preferential metabolic fuel to colonic epithelial cells [17]. Furthermore, SCFAs function as signalling molecules to give rise to a broad range of biological effects in the colonic epithelium, the submucosa and the periphery. One of these functions is the epigenetic regulation of host gene expression via histone deacetylase (HDAC) inhibition [18].

Histone deacetylase enzymes repress gene expression by removing an acyl group bound to chromatin resulting in a tight complex. The overexpression of different isoforms of HDACs has been found in several types of cancer cells as well as in neurological and inflammatory pathologies [19]. In humans, there are a total of 13 HDACs, which are categorised into four main classes—class I (HDACs 1, 2, 3 and 8), class IIa (HDACs 4,5,7 and 9) and class IIb (HDACs 6 and 10), Class III (sirt1-sirt7) and class IV (HDAC 11) [11].

HDAC inhibitors have long been studied in the clinical setting as potential therapeutics [19–23] and there is evidence linking the functional shifts related to microbial-derived HDAC inhibitors and amelioration of disease. In colorectal cancer, for example, an increase in butyrate-producing bacteria prevents cancer cell proliferation via increased histone acetylation [24]. This results in transcription of cancer-related apoptotic genes (BAX, BAK and FAS) [24]. More recently, functional efficacy of the microbial SCFA butyrate as a HDAC inhibitor in colorectal cancer was linked to increased histone crotonylation via inhibition of HDAC2, potentially linking selective HDAC inhibition by the gut microbiota to inhibition of tumorigenesis [25]. Additionally, non-microbially derived valproic acid has been associated with class I HDAC inhibition and amelioration of colitis in a DSS-colitis murine model [3]. This study suggested a role for HDAC class I inhibitors in IFN-γ, IL-10, IL-1β and TNF-α cytokine suppression, assigning functionality to HDAC inhibition and efficacy in colitis [3]. In neurodegenerative disease, sodium butyrate as an HDAC inhibitor has been associated with improvement of motor function in Huntington’s Disease [26]. HDAC inhibitors have also been linked with decreased α-synuclein toxicity in a Parkinson’s Disease (PD) *Drosophila* model [27]. Research is ongoing to find new molecules that inhibit specific HDAC isoforms and their selective role in disease [28].

The gut microbiota, with its immense diversity and metabolic capacity, represents a huge metabolic reservoir for production of a vast variety of molecules with potential effects on HDAC activity. Few studies have assessed the inhibitory effects on HDAC activity of microbial-derived metabolites other than butyrate e.g. medium-chain fatty acids (MCFA), or accumulative effects of different bacterial metabolites on HDAC activity. In the present study, we screened 79 commensal human gut bacteria for their potential global and specific HDAC inhibiting properties *in vitro*, to assess their potential as therapeutic agents due to their selective HDAC inhibitory profiles.

## Materials and methods

### Bacterial culture and cell-free supernatant collection

Pure cultures of 79 phylogenetically diverse bacterial strains from 19 different genera, previously isolated from human faecal samples of healthy donors, were selected from the 4D Pharma Research Ltd. culture collection and grown anaerobically in YCFA broth [Per litre: Casein hydrolysate 10.0 g, Yeast Extract 2.5 g, Sodium hydrogen carbonate 4.0 g, Glucose 2.0 g, Cellobiose 2.0 g, Soluble starch 2.0 g, Di-potassium hydrogen phosphate 0.45 g, Potassium di-hydrogen phosphate 0.45 g, Resazurin 0.001 g, L-Cysteine HCl 1.0 g, Ammonium sulphate 0.9 g, Sodium chloride 0.9 g, Magnesium sulphate 0.09 g, Calcium chloride 0.09 g, Haemin 0.01 g, SCFA 3.1 ml (Acetic acid 2.026 ml/L, Propionic acid 0.715 ml/L, n-Valeric acid 0.119 ml/L, Iso-Valeric acid 0.119 ml/L, Iso-Butyric acid 0.119 ml/L), vitamin mix 1: 1 ml (Biotin 1mg/100 ml, Cyanocobalamine 1mg/100 ml, p-Aminobenzoic acid 3mg/100 ml, Pyridoxine 15mg/100 ml), vitamin mix 2: 1 ml (Thiamine 5mg/100 ml, Riboflavin 5mg/100 ml), vitamin mix 3: 1 ml (Folic acid 5mg/100 ml)] until they reached their stationary growth phase. Cultures were centrifuged at 5000 x g for 5 minutes and the cell-free supernatant (CFS) was filtered using a 0.2 μM filter (Millipore, UK), after which 1 mL aliquots of the CFS were stored at -80 °C until use.

### Ethics

Ethical approval for collection of faecal samples from healthy human donors was granted from the West of Scotland Research Ethics Committee (Ref. **15/WS/0277**). The biological samples have been obtained with any necessary informed written consent from the volunteering participants.

### Cell culture and bacterial CFS treatment

HT-29 human colorectal adenocarcinoma cells were obtained from the European Collection of Cell Cultures (ECACC) (passage 162–173). Cells were grown in Dulbecco’s minimum essential media (DMEM) media containing 10% FBS, 4 mM L-glutamine, 1% non-essential amino acids and antimycotic and antibiotic (Sigma, UK). Three days post-confluence, cells were washed twice with Hank’s Balanced Salt Solution (HBSS) and stepped down in 1 mL of DMEM with 4mM L-glutamine, 1% non-essential amino acids, 5 μg/ml apo-transferrin and 0.2 μg/ml sodium selenite (Sigma Aldrich, UK). Cells were stepped down 24h prior to commencement of the experiment. For the treatment with CFS, 100 μL of DMEM, apo-transferrin and sodium selenite mix were removed from each well, replaced with 100 μL of CFS, and incubated in a CO_2_ incubator for 48 h prior to nuclear protein extraction.

For the treatments with pure SCFA and MCFA, dilutions of sodium butyrate, valeric acid and hexanoic acid (Sigma Aldrich, UK) were prepared in YCFA broth and incubated with HT-29 cells as described above.

### Nuclear protein extraction and total HDAC activity analysis

For nuclear protein extraction, HT-29 cells treated for 48 h with 10% of the different CFS preparations were washed twice with PBS and then harvested by scraping the cells from the wells. Cells were centrifuged at 450 x g for 5 min. Nuclear extractions were then conducted according to manufacturer’s instructions using the NXTRACT NuCLEAR kit (Sigma Aldrich, UK). Once extracted, the nuclear proteins were snap-frozen and stored at -80 °C for HDAC activity analysis. HDAC activity was analysed using the histone deacetylase assay kit (Sigma Aldrich, UK). The assay was conducted according to manufacturer’s instructions using 15 μL of extracted HT-29 nuclear protein.

Additionally, HT-29 nuclear protein of untreated cells was extracted and normalised to the protein concentration of a HeLa cell lysate provided with the NXTRACT NuCLEAR kit (Sigma Aldrich, UK). Protein concentrations were determined using the Pierce Bicinchoninic Protein Assay (BCA) kit A (Thermo Fisher, UK). 15 μL of this extract was used for HDAC activity analysis after incubation of 10% CFS, or dilutions of SCFA (sodium butyrate, valeric acid and hexanoic acid) to confirm HDAC inhibition in whole cells.

### Specific HDAC activity analysis

Specific HDAC inhibition activity was analysed for class I HDACs (1, 2, 3) and class II HDACs (4, 5, 6, 9) using fluorogenic assay kits for each isoform of HDAC (BPS Bioscience, CA). CFS, or dilutions of sodium butyrate, valeric acid and hexanoic acid were diluted 10-fold and exposed to specific HDAC proteins provided in the kit. Assays were conducted according to manufacturer’s instructions and all sample measurements were performed in triplicate.

### SCFA and MCFA quantification of bacterial supernatants

Short chain fatty acids (SCFAs) and medium chain fatty acids (MCFAs) from bacterial supernatants were analysed and quantified by MS Omics APS, Denmark. Samples were acidified using hydrochloride acid, and deuterium labelled internal standards were added. All samples were analyzed in a randomized order. Analysis was performed using a high polarity column (Zebron^™^ ZB-FFAP, GC Cap. Column 30 m x 0.25 mm x 0.25 μm) installed in a gas chromatograph (7890B, Agilent) coupled with a quadropole detector (59977B, Agilent). The system was controlled by ChemStation (Agilent). Raw data was converted to netCDF format using Chemstation (Agilent), before the data was imported and processed in Matlab R2014b (Mathworks, Inc.) using the PARADISe software described by Johnsen *et al*. (2017) [29].

### Culturing of lead candidate strain in an established simplified microbiota consortium

To establish whether HDAC inhibitory effects of a single strain (*Megasphaera massiliensis* MRx0029) could be maintained in an established bacterial community, a defined simplified microbial consortium (SimMi) was designed in an anaerobic continuous culturing system. This system can be extended to several vessels for parallel experiments. The bacterial core consortium is composed of 17 commensal bacterial strains belonging to genera including *Escherichia*, *Faecalibacterium*, *Prevotella*, *Bifidobacterium*, *Bacteroides*, *Lactobacillus*, *Blautia*, *Clostridium*, *Roseburia*, and *Eubacterium*, previously isolated from human faecal samples of healthy donors, and was designed as an in-house model to mimic the main metabolic activity of the human gut microbiota. It covers a wide range of metabolic pathways, mainly focussed on SCFA production, but also considers cross-feeding, bacterial abundance and diversity. The core consortium community was inoculated and allowed to establish for 1 hour prior to a 1% inoculation with the candidate strain *Megasphaera massiliensis* MRx0029. SimMi with and without the candidate strain were run in parallel for 13 days. The metabolism of this consortia versus the control consortium without *M*. *massiliensis* MRx0029 was analysed over 13 days. CFS for HDAC activity analyses were prepared as described above.

## Results

### Gut commensals inhibit total HDAC activity in whole HT-29 cells and on HT-29 cell lysate

**Fig 1:**
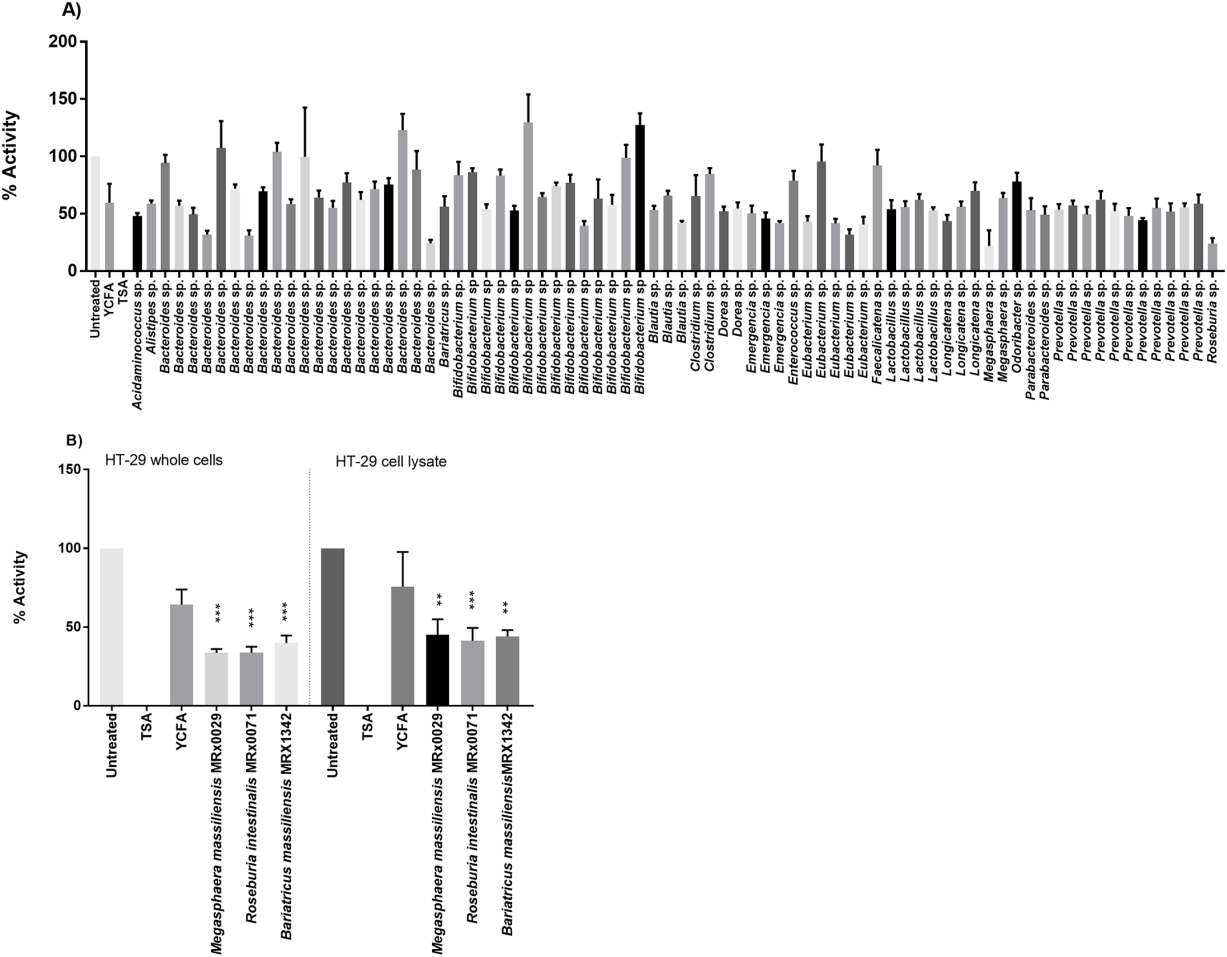
Total HDAC inhibitory effects of supernatants from gut bacterial strains. (A) Screening of cell free supernatants (CFS) from 79 bacterial strains for total HDAC inhibition on whole HT-29 cells. Trichostatin A (TSA) is a negative control. (B) CFS of three selected bacterial strains tested for HDAC inhibition on HT-29 whole cell and HT-29 cell lysates. Trichostatin A (TSA) is used as a negative control. Significances tested against YCFA ** (p<0.005) *** (P<0.001).

The initial screening of the cell-free supernatants of 79 bacterial strains for total HDAC inhibitory effects on HT-29 whole cells resulted in the identification of potential HDAC inhibiting bacterial strains (Fig 1A). The three strains with the strongest HDAC inhibitory effect were identified as *Megasphaera massiliensis* MRx0029, *Roseburia intestinalis* MRx0071, and *Bariatricus massiliensis* MRx1342. CFS of these selected strains were tested again to confirm their HDAC inhibition in HT-29 whole cells and on HT-29 cell lysate to confirm that the HDAC inhibition was not a result of the treatment of the cells prior to nuclear protein extraction. The results in Fig 1B show a similar HDAC inhibition of the supernatants on HT-29 cell lysates as compared to HT-29 whole cells.

### *Megasphaera massiliensis* MRx0029 is the only HDI strain that produces valeric acid

**Fig 2:**
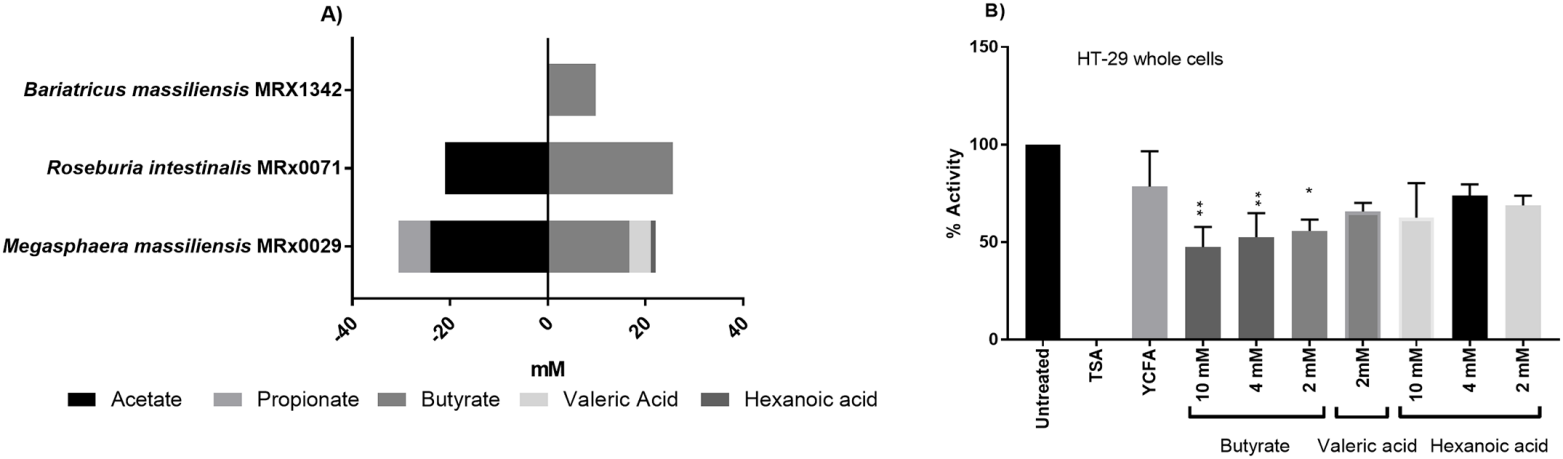
HDAC inhibition of supernatant from gut commensals is primarily driven by butyrate and valeric acid. (A) SCFA and MCFA production of three selected bacteria from screening panel. Strains were grown until stationary growth phase before SCFA and MCFA were measured in CFS. (B) Total HDI in HT-29 whole cells and cell lysate using 10 mM, 4 mM and 2 mM of butyrate, valeric and hexanoic acid. Trichostatin A (TSA) is used as a negative control. Significances tested against YCFA * (p<0.05) ** (p<0.005).

Supernatant analysis for bacterial metabolites of the three candidate strains, i.e. SCFA and MCFA, is shown in Fig 2A. All three bacterial strains, *M*. *massiliensis* MRx0029, *R*. *intestinalis* MRx0071 and *B*. *massiliensis* MRx1342 produced butyrate. *M*. *massiliensis* MRx0029, the strain whose supernatant showed the strongest HDAC inhibition, was the only strain which produced valeric acid and the MCFA hexanoic acid, with a mean concentration of 4.4 mM and 1.0 mM, respectively. To investigate if the SCFAs and MCFAs were the metabolites responsible for the total HDAC inhibition, different concentrations of sodium butyrate, valeric acid and hexanoic acid were tested for HDI on whole HT-29 cells. The results in Fig 2B show a significant (P<0.05) inhibition of HDAC activity by sodium butyrate on HT-29 whole cells, while hexanoic acid did not have a significant effect with any of the tested concentrations. Valeric acid concentrations above 2 mM were toxic to the HT-29 cells, thus HDI could not be measured. Interestingly, the most potent butyrate producer *R*. *intestinalis* MRx0071 (mean butyrate concentration 25.6 mM) closely matches *M*. *massiliensis* MRx0029 (mean butyrate concentration 16.7 mM and mean valeric acid concentration 4.4 mM) with regard to HDI. This suggests a cumulative effect of butyrate and molecules other than SCFA produced by *M*. *massiliensis* MRx0029 being involved in the observed HDI.

### Testing the lead candidate in a simplified model of the human gut microbiota (SimMi)

A community of 17 human gut bacteria was developed in continuous culture to mimic core metabolic functions of the human gut microbiota. This model community was used to investigate the impact of the single strain on the efficacy of an established bacterial community. In addition, this approach was a useful tool to model the behaviour of the strain in the gut environment.

**Fig 3:**
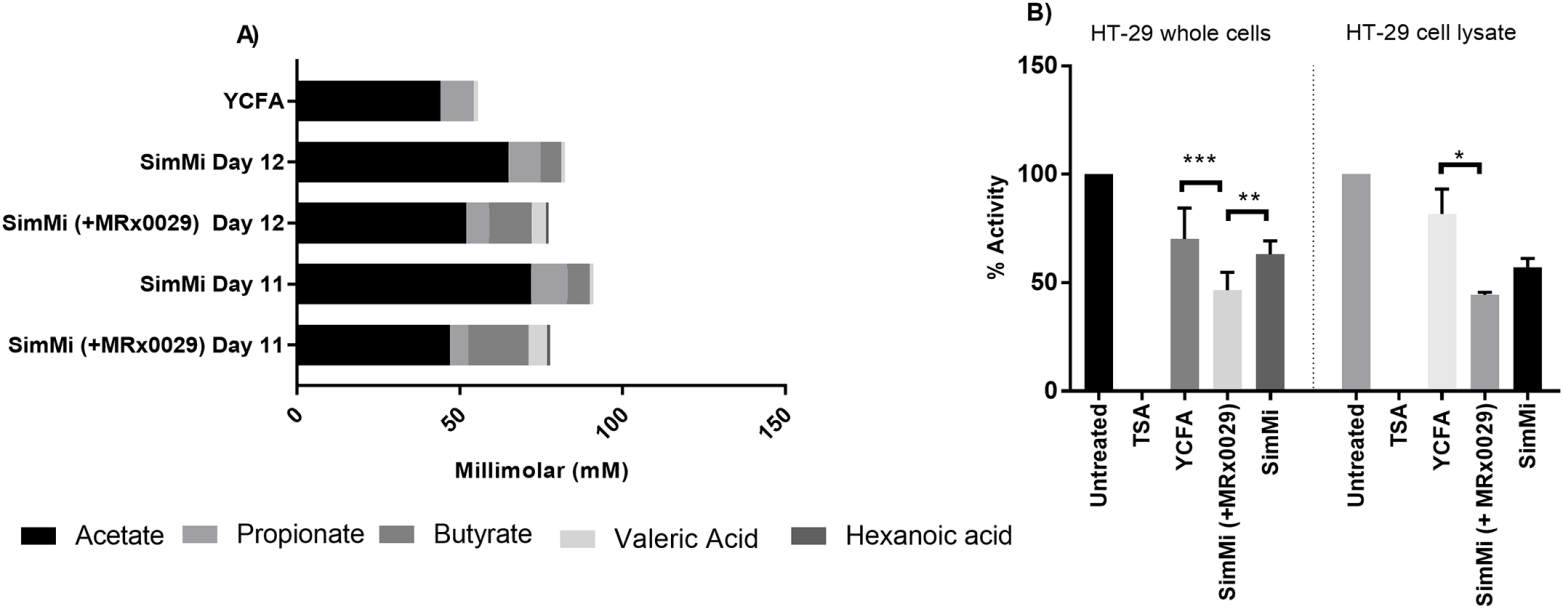
HDAC inhibition of *M*. *massiliensis* MRx0029 is transferable to a microbiota model system (SimMi). (A) SCFA and MCFA concentrations of SimMi consortia (+/- MRx0029) on day 11 and 12 of continuous culture. (B) HDAC inhibition of CFS, obtained from SimMi consortia from day 12 with and without *M*. *massiliensis* MRx0029, on whole HT-29 cells and on HT-29 cell lysate. TSA is used as a negative control. Significances tested against YCFA * (p<0.05) ** (p<0.005) *** (P<0.001).

Valeric acid and hexanoic acid, which were not produced in the original core consortium of SimMi, were produced in the SimMi with added *M*. *massiliensis* MRx0029 over the entire period of the run (13 days) (data not shown), confirming the successful establishment of *M*. *massiliensis* MRx0029 in the SimMi consortium. Samples from two timepoints (days 11 and 12) of SimMi with and without *M*. *massiliensis* MRx0029 were selected and SCFA were measured (Fig 3A). An aliquot from day 11 and 12 of the continuous culture was tested for total HDAC inhibition (Fig 3B) in comparison with supernatants of the core consortium, and blank YCFA broth as control. SCFA profiles in the core consortium on day 11 and 12 were comparable, with concentrations of acetate (65 and 72 mM), propionate (9.9 and 11 mM) and butyrate (6.3 to 6.9 mM). Traces of valeric acid measured were due to the YCFA medium containing this acid. With *M*. *massiliensis* MRx0029 the SCFA profile of the consortium shifted towards higher butyrate concentrations (18.5 and 13.2 mM), and lower acetate (47 and 55 mM) and propionate (5.6 and 7 mM) concentrations than SimMi without *M*. *massiliensis* MRx0029. Additionally, *M*. *massiliensis* MRx0029 led to an increased concentration of valeric acid (5.8 and 7 mM) and low concentrations of hexanoic acid (1.0 and 0.8 mM), which were not produced by the core consortium alone (Fig 3A).

The results from the HDAC activity assay demonstrate that the SimMi consortium with *M*. *massiliensis* MRx0029 exhibited a more potent total HDAC inhibition than the standard consortium on whole HT-29 cells (p<0.001) and on HT-29 cell lysate (p<0.05) (Fig 3B). This demonstrates the physiologically relevant potential of *M*. *massiliensis* MRx0029, as a butyrate and valeric acid producing bacteria, to stimulate HDAC inhibition within an established bacterial community.

### Potent total HDAC inhibitors investigated target class I HDACs

**Fig 4:**
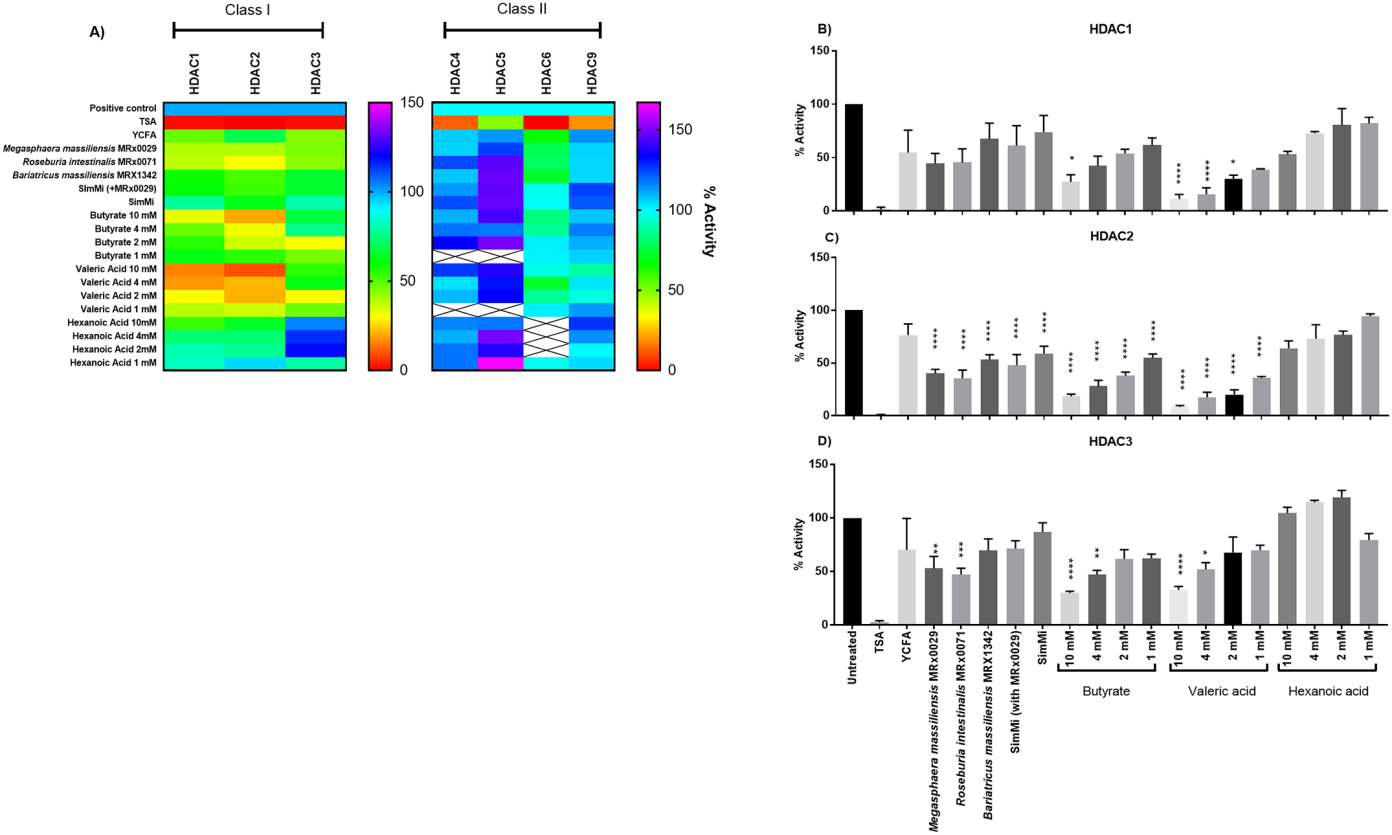
Supernatants of gut commensals selectively inhibit HDAC Class I enzymes—Specifically HDAC2 isoform. (A) Inhibition of different isoforms of class I (HDAC 1, HDAC2 and HDAC3) and class II (HDAC4, HDAC5, HDAC6 and HDAC9) HDACs by CFS of *M*. *massiliensis* MRx0029, *R*. *intestinalis* MRx0071, *B*. *massiliensis* MRx1342, SimMi (+/- *M*. *massiliensis* MRx0029) and dilutions of SCFA butyrate and valeric acid and MCFA hexanoic acid. (B-D) Specific inhibition of HDAC1 (B), HDAC2 (C) and HDAC3 (D) by CFS of bacterial strains *M*. *massiliensis* MRx0029, *R*. *intestinalis* MRx0071 and *B*. *massiliensis* MRx1342, SimMi (+/- *M*. *massiliensis* MRx0029) and different dilutions of SCFA butyrate and valeric acid and MCFA hexanoic acid. TSA is used as a negative control. Significances tested against YCFA * (p<0.05) ** (p<0.005) *** (P<0.001) **** (p<0.0001).

Finally, in suggesting the role of the HDAC inhibitory bacteria in disease, the specific HDAC inhibition profile of the supernatants of the selected bacteria were elucidated. Specific HDAC inhibition assays were carried out for Class I and Class II HDACs using the supernatants of *M*. *massiliensis* MRx0029, *R*. *intestinalis* MRx0071 and *B*. *massiliensis* MRx1342. Furthermore, supernatant samples from day 12 of the SimMi consortium with and without *M*. *massiliensis* MRx0029 were tested. Their abilities to inhibit selected isoforms of HDAC enzymes were compared to different concentrations of butyrate, valeric acid and hexanoic acid. Only the class I isoforms HDAC2 and HDAC3 were inhibited by the CFS and SCFA tested (Fig 4A), no significant inhibitory impact was shown on class II HDACs. Within the class I HDACs the strongest effects were measured for the HDAC2 isoform, where CFS from all three candidate strains as well as SimMi with and without *M*. *massiliensis* MRx0029 resulted in a significant reduction of HDAC2 activity (Fig 4C). The inhibitory effect of SimMi with *M*. *massiliensis* MRx0029 was stronger than the core consortium alone. Sodium butyrate and valeric acid inhibited HDAC2 at all concentrations tested, while hexanoic acid did not show any significant inhibitory effect. HDAC3 was significantly inhibited by *M*. *massiliensis* MRx0029 and *R*. *intestinalis* MRx0071, and only by the higher concentrations of sodium butyrate and valeric acid tested (10 mM and 4 mM) (Fig 4D). HDAC1 was not inhibited by any of the CFS, but by butyrate and valeric acid (Fig 4B). Although not significant, *M*. *massiliensis* MRx0029 and *R*. *intestinalis* MRx0071 consistently showed stronger inhibition against HDAC1, 2 and 3 enzymes compared to MRx1342.

## Discussion

HDACs regulate the acetylation and deacetylation of chromatin strands, leading to variation in DNA expression. Thus, HDAC inhibitors are epigenetic regulators that have pleiotropic effects at cellular and systemic levels. An altered gut microbiota has been associated with diseases such as cancer [30], diabetes [8, 9], asthma [12] and a variety of neurological disorders [31–34], which may be linked to epigenetic aberrations in the host [35]. Screening the CFS of 79 phylogenetic diverse bacteria derived from the human gut microbiota, we have identified several bacteria which produce metabolites that inhibit total HDAC. All strains with this inhibitory effect were butyrate producers. Butyrate is a known inhibitor of class I and class II HDACs [36]. Propionate also possesses this inhibitory effect [37], however, none of the propionate-producing strains from our screening panel showed a strong HDAC inhibition. Butyrate has a multitude of host benefits [18], and is responsible for anti-inflammatory effects not only within the gut, but also systemically, affecting even the brain via the blood-brain barrier [7, 38]. The strain with the strongest inhibitory effect towards HDAC, *M*. *massiliensis* MRx0029, was the only strain in our screening panel which produced amounts of valeric acid (C5) and hexanoic acid (C6). When tested as pure substances, butyrate and valeric acid resulted in significant HDAC inhibition at comparable concentrations produced by *M*. *massiliensis* MRx0029 and *R*. *intestinalis* MRx0071, whilst hexanoic acid exhibited no significant effect. HDAC inhibition activity of several bacterial strains could be attributed to their production of butyrate, but as evident from our results, this SCFA is not the only metabolite with HDAC inhibitory effects. The stronger HDAC inhibition by *M*. *massiliensis* MRx0029 supernatants compared to *R*. *intestinalis* MRx0071 supernatants suggests a cumulative effect of different SCFAs on HDAC inhibition, as a concentration-dependent response was shown for both butyrate and valeric acid. While butyrate has been studied extensively, very little literature describes the therapeutic potential of valeric acid [39]. Our results from total HDAC inhibition showed a much stronger effect for *M*. *massiliensis* MRX0029 and *R*. *intestinalis* MRx0071 than for butyrate or valeric acid alone. It therefore cannot be ruled out that metabolites other than SCFA are produced which contribute to the HDAC inhibitory effects. Other microbial-derived HDAC inhibitors have shown to be selective for HDAC Class I enzymes. Romidepsin (FK228), isolated from *Chromobacterium violaceum* no. 968, and thailandepsin A (TDP-A) and thailandepsin B (TDP-B) discovered from *Burkholderia thailandensis* are now in preclinical and clinical studies for T-cell lymphomas and ovarian cancer [21–23, 40]. Further studies involving untargeted and targeted metabolomics will be necessary to investigate the roles played by other metabolites as potential HDAC inhibitors.

Interestingly, the results for specific HDAC activity of our three lead candidates show that *M*. *massiliensis* MRx0029, *R*. *intestinalis* MRx0071 and *B*. *massiliensis* MRx1342 are potent inhibitors of Class I HDACs, and particularly HDAC2. Class I HDACs (HDAC1, 2, 3 and 8) are small molecules (<500 amino acids) present in the nucleus and are ubiquitously expressed in several human cell lines. HDACs 1–3 share more than 50% homology, but have distinct structures and cellular functions [41]. They are primarily involved in cell survival, proliferation and differentiation, thus being an active player in cancer and inflammatory diseases, including ulcerative colitis [3, 42–45], and a recent study found that the isoform HDAC2 is a crucial target for functional recovery from stroke [46].

When *M*. *massiliensis* MRx0029, one of the strains with the strongest HDAC inhibitory effect, was added to a simplified human gut microbiota consortium, its strong inhibitory effects of the pure culture were transferred to the bacterial community. The SimMi consortium with *M*. *massiliensis* MRx0029 showed a significantly stronger HDAC inhibition when compared to the original core consortium. This implies that the production of valeric acid, as well as the higher butyrate production after the addition of *M*. *massiliensis* MRx0029 were at least partly responsible for these effects. Similar results were obtained for specific HDAC2 and HDAC3 inhibition. It clearly indicates that our candidate strain MRx0029 is producing metabolites that are potent HDAC class I inhibitors, extending the activity to an entire community as evidenced by the SimMi consortium results. Our findings make MRX0029 a promising candidate as a live biotherapeutic strain for disease areas involving epigenetic aberrations, via inhibition of HDAC2 activity. *M*. *massiliensis* MRx0029 mode of action needs to be investigated by testing it as a biotherapeutic strain in specific disease models, in which class I HDAC inhibition plays a role.

The results from our study could thus lead to development of live biotherapeutics which target specific epigenetic regulators of diseases.

The funder provided support in the form of salaries for authors [SY, NR, HD, SP, IEM], but did not have any additional role in the study design, data collection and analysis, decision to publish, or preparation of the manuscript. The specific roles of these authors are articulated in the ‘author contributions’ section.

## Supporting information

S1 Data Raw data file. (XLSX)
